# Identification of Hollow Viscus Injury with FAST Examination in Kurdistan, Iraq

**DOI:** 10.1155/2018/5019415

**Published:** 2018-02-13

**Authors:** Ruj Al-Sindy, Heleen Alaqrawy, Mahmood Sh. Hafdullah, Christine Butts

**Affiliations:** ^1^Emergency Hospital, Duhok, Kurdistan, Iraq; ^2^Section of Emergency Medicine, Louisiana State University, New Orleans, LA, USA

## Abstract

Point-of-care ultrasound has become indispensable in the evaluation of trauma, particularly in low resource areas, where it may be the only rapidly available imaging modality. The FAST (Focused Assessment with Sonography in Trauma) in particular can be lifesaving, by rapidly detecting signs of intra-abdominal hemorrhage. However, the FAST is primarily designed to identify free fluid associated with solid organ injury and is thought to have less sensitivity and power in identifying evidence of hollow viscus injury. We present a case of an unidentified man that presented to a hospital in the Kurdistan region of northern Iraq, a region of low resources, surrounded by war. The FAST exam proved to be the key to identifying this patient's injuries.

## 1. Background

Although hollow viscus injuries resulting from blunt abdominal trauma (BAT) are fortunately rare, they convey a high morbidity and mortality. Diagnosing these injuries remains challenging, despite advances in imaging. The FAST examination (Focused Assessment with Sonography in Trauma) has been extensively studied as a tool in the evaluation of free fluid resulting from solid organ injury, but less so for the more subtle findings of hollow viscus injury. FAST examination may be the only immediately available imaging modality in low resource environments, and the determination of characteristics that could rapidly identify findings of viscus injury could prove invaluable. We present a case from Kurdistan, Iraq, in which a bedside FAST examination identified a suspected and later confirmed case of ruptured viscus.

## 2. Case Report

An approximately 30-year-old male was brought to the Emergency Hospital in Duhok, a city of 350,000 in the Kurdistan region of northern Iraq. The Kurdish military, the Peshmerga, who had found the patient in the woods, brought the patient to the emergency department. The patient was unable to give any history on arrival due to his condition. However, the Peshmerga related that he had reportedly been held by a Shia militia as a suspected Islamic State terrorist for approximately 20 days and had been tortured and starved. He had managed to escape and had been hiding in the woods, subsisting on grass, for 15 days prior to being found and brought to the ED.

**Figure 1:**
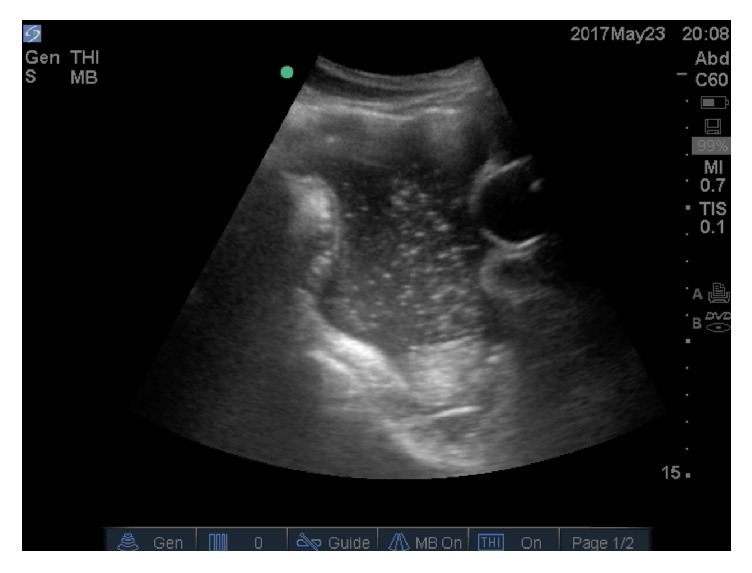
Longitudinal image of free fluid in the pelvis. Particulate matter is seen within the free fluid. The circular balloon of the urinary catheter can be seen on the right of the image.

The patient's initial vital signs were significant for tachycardia at a rate of 200, an undetectable blood pressure, and an oxygen saturation of 43% on room air. A temperature was not initially assessed but was subsequently noted to be 36°C. Multiple large bore intravenous catheters were placed immediately with initiation of fluids. He was unresponsive with a GCS of 3 and was intubated in anticipation of an inability to protect his airway. His primary survey was also significant for decreased breath sounds bilaterally. Due to the level of hypoxia, pneumothorax was considered highly likely, and a bedside ultrasound was performed to confirm bilateral pneumothorax. After needle decompression, bilateral chest tubes were placed. The patient's abdomen was distended, but not rigid. Tenderness could not be assessed as the patient was GCS 3 and unresponsive. No immediately obvious source of hemorrhage was noted, but his blood pressure continued to be low, and intra-abdominal injury was highly suspected. A FAST examination was performed, which demonstrated complex free fluid in all quadrants. Particulate matter was noted within the free fluid, most notably in the pelvis (Figure 1).

**Figure 2:**
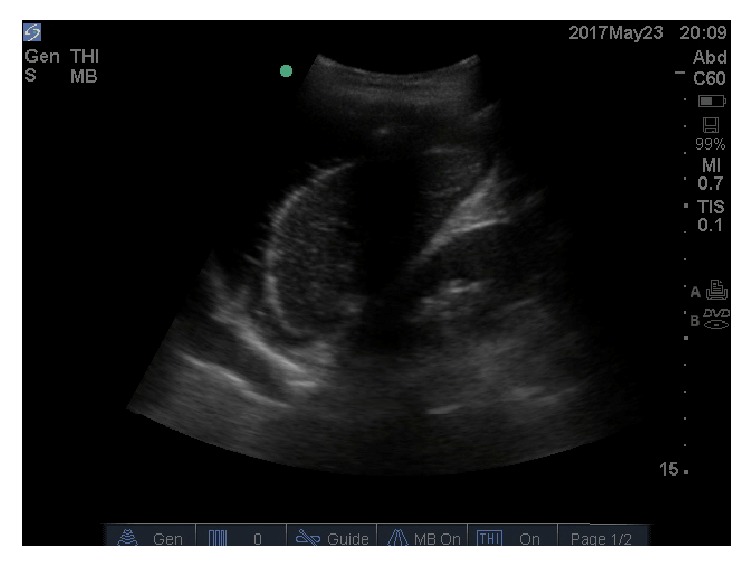
Longitudinal image of the left upper quadrant. Fluid can be seen not only surrounding the spleen, but also superior to the diaphragm on the far left of the image.

Fluid was also noted to the left pleural space (Figure 2).

**Figure 3:**
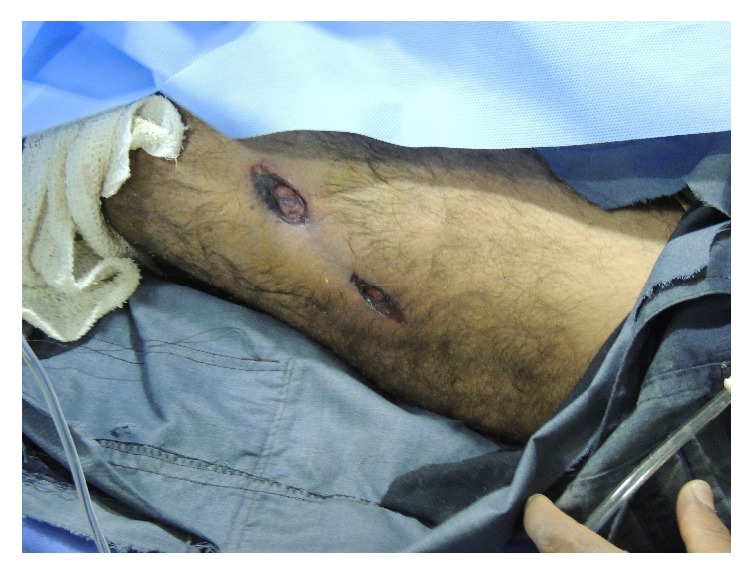
Lesion noted to the left anterior thigh with charred edges, possibly representing a burn.

The secondary survey further revealed diffuse bruising of varying ages over the patient's extremities and torso. Multiple skin defects were noted to the lateral left thigh, which had charred edges consistent with a burn (Figure 3).

**Figure 4:**
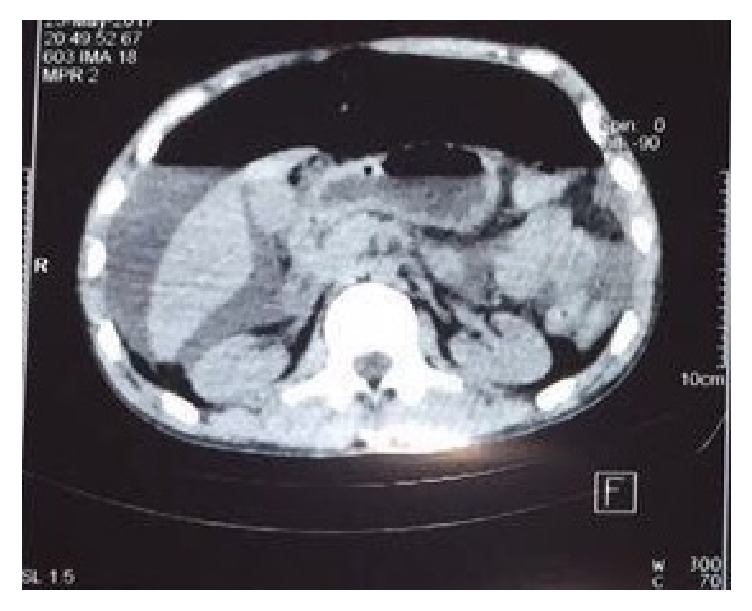
CT abdomen image demonstrating free fluid to the right upper quadrant and free air.

Following the secondary survey, the initiation of broad-spectrum antibiotics, and the placement of a urinary catheter and orogastric tube, the patient was transported to CT for further evaluation. A head CT was unremarkable for acute findings. The chest CT confirmed the diagnosis of bilateral pneumothorax, while the abdominal CT demonstrated a large amount of free fluid and air within the abdomen and pelvis (Figure 4). No obvious source for these findings was identified on the CT. There were no obvious solid organ or hollow viscus injuries. The patient's blood work demonstrated the following significant findings: white blood cell count of 3,400 per microliter, a creatinine of 5 mg/dl, a BUN of 263 mg/dl, AST of 277 mg/dl, ALT of 155 mg/dl, total bilirubin of 2.11 mg/dl, and an amylase of 126 U/l. A blood gas showed the patient's pH to be 7.29, with adequate oxygenation.

The general surgeon on call evaluated the patient and felt that he was not a good candidate for surgery, given his hemodynamic instability and the limited resources. At this point in the ongoing battle with the Islamic State, the Emergency Hospital in Duhok was receiving large numbers of casualties per day, far outpacing their available resources. Unfortunately, although this hospital has limited resources, it is the most advanced hospital in the area. Also, due to the danger of the ongoing conflict in the area and lack of an organized prehospital medical system, transfer to a more advanced hospital was not a viable option. A drain was placed into the abdomen, which drained over 3 liters of bilious material. Unfortunately, despite the treatments he received, the patient died 3 days after presentation.

## 3. Discussion

Blunt abdominal trauma (BAT) is frequently encountered in the emergency department, whether it occurs due to motor vehicle collision (MVC), fall, or other mechanism. The prevalence of intra-abdominal injury encountered in these patients is substantial, and the mortality and morbidity conveyed by these injuries are significant [1]. Within the larger group of BAT, hollow viscus injuries are fortunately uncommon [2]. One recent study evaluated over 3500 patients presenting with BAT. They found that of the 285 that had an intra-abdominal injury, only 3.2% had an isolated hollow viscus injury, although 14 patients had a solid organ injury in addition to a hollow viscus injury [3]. Although hollow viscus injury is rare, it is an important diagnosis to consider, as it is associated with high morbidity consequences, such as sepsis [4]. Early consideration is the key to preventing complications, as a delay in diagnosis is associated with increased morbidity [5].

Though hollow viscus injury is a critical diagnosis to make, identifying it is not straightforward. Many patients may have neurologic injuries or intoxicants that alter their level of consciousness and their physical examination may be unreliable. In one study nearly 20% of patients had unremarkable abdominal physical exams in the face of positive findings on CT scan [6].

CT scan, which is commonly used in the evaluation of patients suffering BAT, has shown mixed results in making the diagnosis of hollow viscus injury. Sensitivities for small bowel injury range from 83% to 94%, with accuracies of 84% to 99% [2]. CT findings of intraperitoneal gas, intramural air, extraluminal oral contrast, extraluminal intestinal contents, and disruption to the bowel wall are highly suggestive of hollow viscus injury [7, 8]. However, these findings may be present in only a minority of cases [2]. Frequently, other more subtle findings, such as free fluid in the absence of solid organ injury, may be the only clue to the diagnosis [9]. When solid organ injuries are concomitantly present, the diagnosis may be even more difficult.

Ultrasound in trauma relies upon the primary detection of free fluid, which may not be present, or may be present in only miniscule amounts, in hollow viscus injury [10]. The performance of the FAST examination in the diagnosis of these injuries thus far has primarily been evaluated as part of larger studies of BAT. Limited available evidence shows mixed results, with reported sensitivities ranging anywhere from a dismal 42% to a much better 88%. Specificities are higher, 98–100% [1]. Although the FAST has been well studied as a component of the evaluation of BAT and is included in management guidelines, its specific use as a focused tool in the diagnosis of hollow viscus injury has not been extensively studied and high level recommendations for this use do not exist [11].

Although the evidence for the use of the FAST in evaluating patients with suspected hollow viscus injury is lacking overall, bedside ultrasound is commonly used in the evaluation of trauma patients, particularly in low resource environments. In these areas, CT scan may not be available or access may be delayed, and many low resource environments may lack more advanced, higher resolution scanners. Additionally, in areas where the volume of injured patients is overwhelming, such as in war zones, FAST may be the most practical and efficient way of evaluating and triaging a large number of casualties. Determining which patients receive operative intervention or CT scan and in which order may depend on the results of the FAST examination.

In the case highlighted above, significant intra-abdominal trauma was immediately suspected due to the patient's hemodynamic instability. Once other sources of possible shock, such as pneumothorax, were identified and treated, the persistence of instability made the FAST examination even more critical. The identification of complex free fluid with particulate matter was immediately suspicious for hollow viscus injury for the treating physicians. CT scan seemed to add additional evidence for this diagnosis, as free fluid with no evidence of solid organ injury was found. Aspiration of intestinal contents by the treating surgeon appears to confirm the diagnosis.

Although no specific guidelines exist for the use of FAST examination in the diagnosis of hollow viscus injury, this case highlights findings that are suggestive and may add substantially to clinical suspicion.
